# Successful Orthograde Treatment of Teeth with External Inflammatory Root Resorption and Perforation Using a Tricalcium Silicate-Based Material

**DOI:** 10.1155/2022/7119172

**Published:** 2022-12-30

**Authors:** Mariana Díaz, Hugo Plascencia, Fabián Esaú Hernández-Guerrero, Flávio R. F. Alves, Karina Martínez-Carrillo, Gerardo Gascón

**Affiliations:** ^1^Endodontic Postgraduate Program, University Center of Health Sciences (CUCS), University of Guadalajara, Guadalajara, Mexico; ^2^Department of Endodontics and Dental Research Group, Iguaçu University, Nova Iguaçu, Rio de Janeiro, Brazil; ^3^Postgraduate Program in Dentistry, University of Grande Rio, Duque de Caxias, Rio de Janeiro, Brazil

## Abstract

One possible consequence of dentoalveolar trauma is the development of external inflammatory root resorption (EIRR), which represents an anatomic and microbiologic challenge for clinicians. This case report describes different strategies implemented for successful endodontic management of teeth with multiple EIRR lesions, highlighting the orthograde root canal filling using a tricalcium silicate-based material (Biodentine, Septodont, Saint-Maur-des-Fossés, France). A 17-year-old female patient presented with severe pain in the anterior maxillary teeth and a history of trauma. Two- and three-dimensional radiographic exams confirmed EIRR in three teeth, with a total of 11 EIRR lesions, three exhibiting communication with the root canal. Therefore, chemo-mechanical preparation complemented by ultrasonic activation of irrigants and some changes of intra-canal dressing with calcium hydroxide were performed to reduce the microbiologic load of the affected teeth as much as possible. Then, the canals were entirely filled with Biodentine to interrupt the root resorption process and strengthen the remaining root structure. A 60-month follow-up showed the disappearance of bone rarefactions and the complete repair of the 11 EIRR lesions. The favorable long-term response indicates the feasibility of using tricalcium silicate-based putty as part of orthograde endodontic treatment of teeth with EIRR and root perforations.

## 1. Introduction

External inflammatory root resorption (EIRR) is a pathologic after-effect of dental trauma, characterized by the loss of root structure and periradicular radiolucencies [1]. Although its etiopathogenesis is not well understood, a combination of several factors is required for its establishment. Initially, significant dentoalveolar trauma must mechanically damage and ‘carve' several areas of the tooth-supporting hard tissues. As part of the healing process, macrophages and osteoclasts remove the injured tissues and expose different regions of the root dentin, initiating a generally self-limiting external root resorption [2]. However, an endodontic infection can concurrently occur in the pulp space due to untreated or inadequately treated trauma. Furthermore, communications between the root canal and the periapical tissues could be established if the exposed dentin has wide and permeable dentinal tubules. Thus, microorganisms and their products could invade the affected area and stimulate osteoclast activity or exacerbate a pre-existing one [3]. This concern is greater in younger patients with roots in the early stages of development (Cvek stages I, II, and III) because the root canal walls are thinner, and the passage of microbial irritants is facilitated [4].

In response to the dental trauma associated with the root canal infection, osteoclasts and immune cells initiate the demineralization and progressive phagocytosis of the dental hard tissues, creating small resorption lacunae that are invaded by granulomatous tissue [5]. The EIRR is formally established at this stage. Small EIRR lesions have an abrupt and accelerated growth, and radiolucent excavations become radiographically visible along the root surface, with rarefactions in the adjacent alveolar bone [1]. If the reabsorption is not interrupted, it progresses and can communicate with the root canal, significantly weakening the root structure or even leading to complete root resorption within a few months [6]. Besides, the possible infection of the resorption lacunae by microorganisms arising from the root canal can compromise the orthograde root canal treatment success [7]. In context, there is no unique protocol to treat the EIRR, and its management depends on the characteristics of each case [8].

Tricalcium silicate-based materials are inorganic products that are strongly compatible with periapical tissues and have revolutionized the management of numerous clinical scenarios in endodontics [9, 10]. Some of the unique benefits include inducing the formation of new alveolar bone, cementum, and periodontal ligament (even allowing their adhesion on the surface of the material) [11]. These types of cement also deposit hydroxyapatite in pores and material/dentin interfaces [12, 13], thus creating a compact and homogeneous monoblock. However, the long-term behavior of such products applied in cases of resorption lesions that weaken the root structure and communicate with the pulp space is unknown. The present case report aimed to describe the successful endodontic management of three teeth with multiple EIRR lesions, including some communicating with the root canal, by orthograde filling using a tricalcium silicate-based material.

## 2. Case Presentation

A 17-year-old female patient was attended with a complaint of intense nocturnal pain in tooth numbers 11 (maxillary right central incisor), 21 (maxillary left central incisor), and 22 (maxillary left lateral incisor). The pain developed over two days. The dental history revealed a trauma that occurred 10 months before (a bicycle fall), with slight posterior displacement of the involved teeth and intense gingival bleeding, indicating lateral luxation in the three teeth. According to the report, the patient's mother repositioned the teeth immediately after the accident and instructed her daughter to keep her mouth closed for 30 minutes. As the bleeding ceased and the teeth apparently returned to their original position, the patient did not visit a dentist at that moment. The medical history revealed no previous systemic problems or allergies.

**Figure 1:**
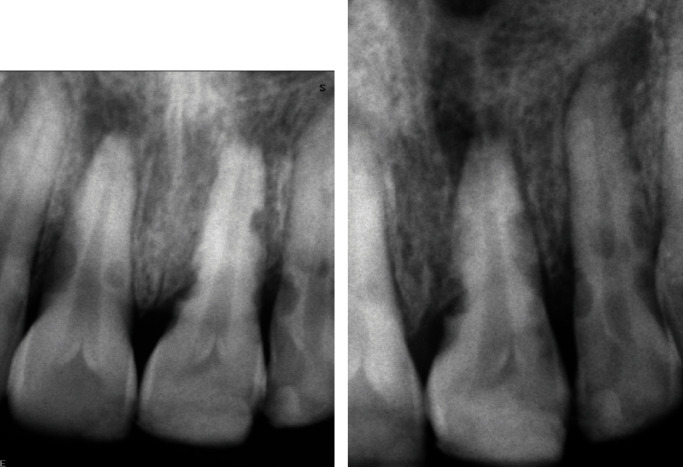
Pre-operative radiographs showing multiple external inflammatory root resorption lesions. (a) Teeth number 11 and 21. (b) Teeth number 21 and 22.

The clinical examination showed that the crowns did not present any color changes, and there was no inflammation or discoloration of the mucosa. Tooth numbers 11, 21, and 22 were extremely tender to percussion and palpation. Cold (Endo-Ice™; Hygenic Corp., Akron, OH, USA) and electrical (Digitest; Parkell Inc., Farmingdale, NY, USA) sensitivity tests were negative and contrasted with the normal responses of the neighboring teeth. Probing depth and mobility were within normal limits. The three traumatized teeth showed similar characteristics in the periapical radiographic analysis; the pulp chambers were normal, with straight roots and closed apices. However, apical periodontitis and multiple excavations on the root surfaces could be detected in all affected teeth. The resorption lesions thinned the dentinal walls, which had crater-shaped indentations and showed radiolucent regions in the adjacent bone (Figure 1). After collecting the clinical and radiographic findings, a diagnosis of pulp necrosis and acute apical abscess and EIRR was established for the three affected teeth. Treatment options were presented and discussed with the patient and her mother in terms of prognosis, being the decision for the orthograde root canal treatment taken together.

In the same session, the endodontic treatments were initiated after the patient's parents signed an informed consent form. Three-fourths of one cartridge of 2% mepivacaine (Scandonest with 1 : 100,000 epinephrine; Septodont, Saint-Maur-des-Fossés, France) were applied via local infiltration to each of the three teeth. Once a dental dam and marginal sealing were placed, the pulp chamber was accessed using high-speed burs, #3 carbide ball, and Endo-Z. All procedures were performed with the aid of an operating microscope (Zeiss Opmi; Carl Zeiss, Jena, Germany). The root canal entrances were identified, and asepsis of the operative field was accomplished using gauze soaked in 5.25% sodium hypochlorite (NaOCl). Negotiation of the root canals was achieved using a #15 manual K-file under abundant irrigation with 2.5% NaOCl. By choice, cervical preflaring was not performed to preserve dentin as much as possible. The working length was determined using radiographs and established at 1 mm short of the root apex. Next, light chemo-mechanical brushing of the main canals was performed with rotatory NiTi files (X1 and X2 ProTaper Next; Dentsply Maillefer, Ballaigues, Switzerland), followed by manual instrumentation using balanced forces with #40 K-files. The final apical preparation sizes in tooth numbers 11 and 21 were 80, while in tooth number 22 was 70. Syringe irrigation with a 30G notched open-ended needle was performed using 3 mL of 2.5% NaOCl between each instrument. The needle was always placed at 2 mm short of the working length. Even with gentle irrigation, the patient reported mild pain during the irrigation in the three teeth each time the NaOCl was released. This occurrence indicated a possible extrusion of irrigation solution into the periapical tissues due to the potential presence of EIRR lesions communicating with the main canal. After final irrigation with 5 mL of 17% ethylenediaminetetraacetic acid and ultrasonic activation for 20 seconds, the canals were dried, and intra-canal dressing with a calcium hydroxide paste mixed with saline was applied. Then, the coronal cavity was sealed with temporary cement (Cavit G; 3M ESPE, St Paul, MN, USA). Post-operative instructions were provided, and a 250 mg paracetamol tablet was prescribed every eight hours in case of discomfort.

The patient returned two weeks later without symptoms. Then, a cone-beam computed tomography (CBCT) was requested for the three-dimensional mapping of the lesions in terms of number, location, and size, as well as to confirm the suspected communication with the main canal. First, the intra-canal dressing was removed to not interfere with the CBCT exam. The removal was performed under rigorous aseptic conditions as described for the first session. After coronal sealing removal, the intra-canal dressing was eliminated by abundant irrigation with 2.5% NaOCl and mechanical cleaning with #25 K-files. Each canal was finally irrigated using 5 mL of 2.5% NaOCl and ultrasonically activated for 20 seconds. Unlike during the first appointment, the patient did not report discomfort during the irrigation procedures. The canals were then dried, and the pulp chamber was resealed.

**Figure 2:**
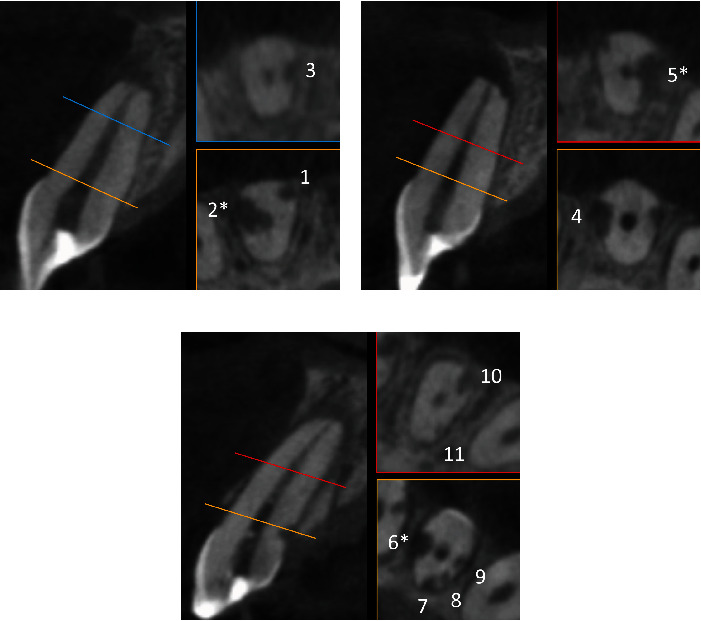
Representative three-dimensional images of external inflammatory root resorption (EIRR) lesions. (a) Tooth number 11 showed three lesions: two in the cervical third (yellow line and box) and one in the middle third (blue line and box). (b) Tooth number 21 showed two lesions: one in the cervical third (yellow line and box) and one in the apical third (red line and box). (c) Tooth number 22 showed six lesions: four in the cervical third (yellow line and box) and two in the apical third (red line and box). ∗Indicates EIRR lesions with root perforation (EIRR #2, #5, and #6).

The following day, the patient returned with the CBCT. The intra-canal dressing was reapplied after the same sequence of anesthesia, isolation, and chemo-mechanical cleaning, and the access cavity was sealed. The CBCT was analyzed by two different observers who measured the height, width, and depth of the EIRR lesions. The means of these measurements are shown in Table 1. A total of 11 EIRR lesions were found and identified by consecutive numbers (Table 1 and Figure 2): three in tooth number 11, two in tooth number 21, and six in tooth number 22. The lesions were predominantly located in the cervical third of the roots (*n* = 8), while some showed evidence of canal perforation (*n* = 3, EIRR #2, EIRR #5, and EIRR #6). Although tooth number 22 had numerous resorption lesions (*n* = 6, EIRR #6–#11), they were all small, circular, and contrasted with some lesions from the other teeth (EIRR #2, EIRR #4, and EIRR #5). EIRR #5 from tooth number 21 showed substantial dimensions and an elongated shape that covered several root thirds. Based on these findings, the initial treatment plan was reinforced by applying strategies focused on creating an environment that would favor the interruption and repair of EIRR lesions by depositing new hard tissue, particularly in teeth with perforations EIRRs.

**Figure 3:**
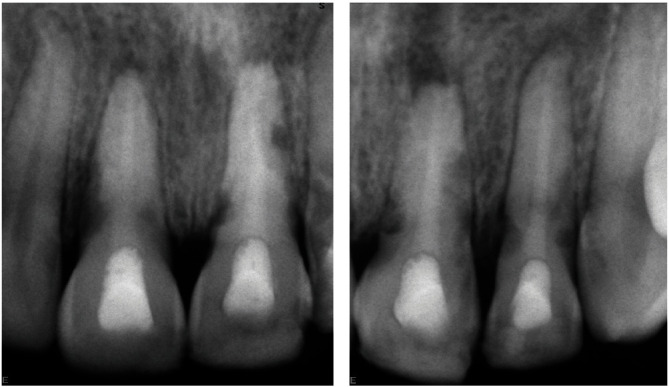
Post-treatment radiographs. (a) Root canal filling in teeth number 11 and 21. (b) Root canal filling in teeth number 21 and 22.

After 30 days, a third intra-canal dressing renewal was performed. Then, the patient was monitored at a distance, and once the patient was asymptomatic for 45 days, the root canal filling was performed. First, the same previously described sequence of anesthesia, teeth isolation, intra-canal dressing removal, and chemo-mechanical cleaning was performed. Due to CBCT findings indicating the presence of three resorption lesions communicating with the pulp space, the root canals were carefully explored with the combined use of the dental operating microscope and DG-16 endodontic explorers (American Eagle, CA, USA). All three suspected perforation lesions were confirmed clinically (EIRR #2, EIRR #5, and EIRR #6). Second, the canals were dried using sterile paper points, and calcium hydroxide powder was inserted using conveyors (MAP System Universal Kit; Dentsply Maillefer, Ballaigues, Switzerland) and compacted with Schilder's pluggers to create a 0.5 mm thick apical barrier against which to pack the root canal filling material in order to prevent its extrusion into the periapical tissues. Third, a tricalcium silicate-based putty (Biodentine; Septodont, Saint-Maur-des-Fossés, France), previously activated according to the manufacturer's recommendations, was inserted (MAP System) and compacted sequentially with Schilder's pluggers until reaching the cementoenamel junction. Finally, the excess material was removed and the access cavity was sealed using composite-based sealing material (FiltekTM Supreme XT; 3M ESPE, St Paul, MN, USA). The final radiograph demonstrated adequate filling of the pulp space (Figure 3).

**Figure 4:**
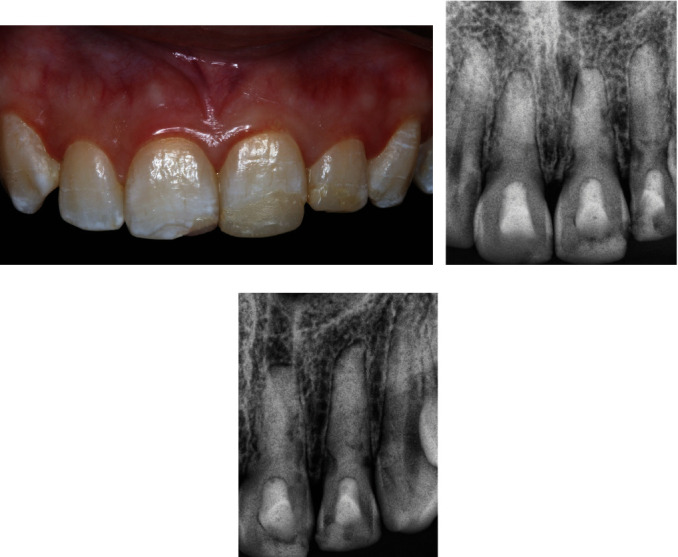
Clinical and radiographic appearance at the 60-month follow-up. (a) Intraoral frontal view of the patient. (b) and (c) Follow-up radiographs.

**Figure 5:**
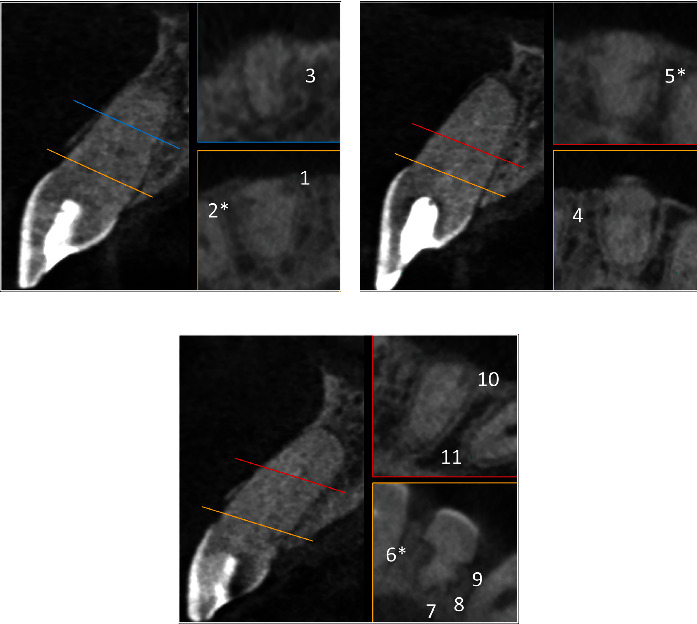
Three-dimensional appearance of the 11 external inflammatory root resorption (EIRR) lesions at the 60-month follow-up revealing adequate healing. (a) Tooth number 11 showed three lesions: two in the cervical third (yellow line and box) and one in the middle third (blue line and box). (b) Tooth number 21 showed two lesions: one in the cervical third (yellow line and box) and one in the apical third (red line and box). (c) Tooth number 22 showed six lesions: four in the cervical third (yellow line and box) and two in the apical third (red line and box). ∗Indicates EIRR lesions with root perforation (EIRR #2, #5, and #6).

At 60 months post-treatment, the patient reported no pain or clinical signs of re-infection. The dental crowns showed no discoloration (Figure 4(a)). Also, probing depths and mobility were within normal limits. There were no findings of ankylosis, that is, infra-occlusion or a metallic sound on percussion. The periapical radiographic analysis confirmed the total disappearance of the apical and lateral radiolucent lesions, with evidence of hard tissue deposition and reestablishment of cortical bone in the three affected incisors (Figures 4(b) and 4(c)). A new CBCT exam was requested, which confirmed the complete repair of all the EIRR lesions, including the eight located in the cervical third of the roots. In addition, the three perforating EIRR lesions were also repaired and showed hard tissue deposition (Figure 5).

## 3. Discussion

The EIRR is a biological response to infection and traumatic dental injuries, and its management represents an anatomic and microbiologic challenge for clinicians. This case report described a conjunct of strategies responsible for the successful orthograde endodontic treatment of three traumatized teeth with multiple EIRR lesions, including some communicating with the root canal.

The diagnostic of EIRR is dependent on radiographic exams. However, detecting resorption lesions by periapical radiographs is only possible in more advanced stages and when they are located in the proximal root surfaces [14]. Therefore, small-field-of-view CBCT is strongly recommended in cases of EIRR not only to identify lesions in early stages but also those located on the buccal or palatal/lingual surfaces, allowing a three-dimension mapping of the affected root surface [15]. This is crucial to establish an individualized treatment plan, as reported in this case.

The treatment strategies adopted in the present case were focused on arresting the resorption process, following recommendations from previous studies [5, 6, 16]. Careful irrigation with NaOCl and periodic changes of intra-canal dressing with calcium hydroxide were performed to reduce the microbial load and increase the pH of the peripheral root dentin as much as possible. The diffusion of hydroxyl ions through the dentinal tubules to the cementless root surface can inhibit clastic cell activity and reduce inflammation of the periodontal ligament [6, 16].

Although the filling material was considered irrelevant to the success of treatment of EIRR in the past [15], calcium silicate-based filling materials have shown promising results in the healing of the tooth-supporting apparatus of teeth with EIRR since they promote a prolonged release of hydroxyl ions [17]. Therefore, they can strengthen the weakened root structure by inducing the deposition of new hard tissue inside the EIRR lesions, particularly in those communicating with the pulp space. Another advantage is that the acidic pH, typical of an endodontic infection, does not affect their properties, including the microhardness.

In the present case report, given that the affected teeth presented resorption defects along the entire root length, a tricalcium silicate-based putty (Biodentine) was used to fill the canals from the apical foramen to the amelo-cemental junction. Previous studies demonstrated the gradual deposition of hydroxyapatite in the material/dentin interface, dentinal tubules, and surrounding intertubular dentin induced by Biodentine [12, 13]. This deposition creates a monoblock that may increase the fracture resistance of the tooth and provides greater long-term predictability to the procedure, as verified in this case.

According to Ricucci et al. [18], once the EIRR is controlled, stem cell recruitment and differentiation are required to repair the lesions in both dentin and root cementum, regardless of their number, extent, or depth. These regions of resorption are exclusively repaired by cementum-like tissue [19, 20]. Therefore, the long-term clinical and radiographic follow-up is critical because cervical root fracture, deep periodontal pocket, and dental ankylosis are potential post-treatment complications. However, the greatest concern following orthograde management of teeth with EIRR is a possible recurrence resulting from the presence of metaplastic cells in the adjacent periodontal ligament that could differentiate into clastic cells [21]. A tricalcium silicate-based putty may prevent the formation of these malformed cells since it allows for the regeneration of the injured root cementum and periodontal ligament [22]. In the present case, it was possible to confirm the repair and maintenance of dental integrity at a 60-month follow-up exam.

There are few *in vivo* studies in which roots with EIRR were filled with tricalcium silicate-based putty, most of them using animal models [23–26] and other some reports in humans [20, 27–30]. However, this is the first report in which a bioactive-based material in putty form was used for the orthograde management of EIRR lesions, including some communicating with the root canal, demonstrating be a safe and reliable approach over the long term. Even so, although the use of a tricalcium silicate-based cement may have promoted the healing of this condition, to affirm that its use was the differential responsible for the successful treatment could be premature. It is important to highlight that this cement was part of a whole clinical strategy based on biological concepts.

Finally, the clinicians must be prepared to face the challenges posed by EIRR, mainly when the reabsorption lesions communicate with the root canals. A careful periapical radiographic and tomographic examination provides insights into the extent of the resorption process and enables the clinicians to establish individualized treatment strategies focused on suppressing the resorption process, eliminating the infection, and strengthening the root structure over the long term. The findings of this case suggest the use of tricalcium silicate cement in the putty form as an important ally in the orthograde endodontic treatment of teeth with EIRR lesions, including those communicating with the root canal. However, non-experienced clinicians should promptly refer patients with this type of condition to a specialist.

## Figures and Tables

**Table 1:** Three-dimensional findings in three maxillary incisors with external inflammatory root resorption (EIRR).

Lesion number	Tooth number	Location		Dimensions (mm)			With perforation
		Third	Dental surface	Height	Width	Depth	
EIRR #1	11	Cervical	Mesio-buccal	2.1	0.9	1.6	No
EIRR #2	11	Cervical	Distal	4.9	2.6	2.8	Yes
EIRR #3	11	Apical	Mesial	2.8	1.2	1.1	No
EIRR #4	21	Cervical/middle	Mesial	3.8	3.2	1.7	No
EIRR #5	21	Cervical/apical	Distal	7.6	2.1	2.4	Yes
EIRR #6	22	Cervical	Mesial	3.2	2.3	1.8	Yes
EIRR #7	22	Cervical	Palatine	1.8	1.3	1.5	No
EIRR #8	22	Cervical	Disto-palatine	1.3	0.9	1.3	No
EIRR #9	22	Cervical	Distal	1.4	1.4	1.1	No
EIRR #10	22	Middle	Disto-buccal	2.8	1.0	1.9	No
EIRR #11	22	Middle	Disto-palatine	0.9	1.1	0.8	No

## Data Availability

The datasets generated during the current study are available from the corresponding author on reasonable request.

## Supplementary Materials

Supplementary Materials CARE CHECKLIST The case description of this manuscript was performed according to the CARE checklist.
