# Supplementary material for: Successful Orthograde Treatment of Teeth with External Inflammatory Root Resorption and Perforation Using a Tricalcium Silicate-Based Material
Source: Case Rep Dent. 2022 Dec 30;2022:7119172. doi: 10.1155/2022/7119172 (PMC9822737; doi:10.1155/2022/7119172)
Supplement: Supplementary Materials — CARE CHECKLIST The case description of this manuscript was performed according to the CARE checklist. [file 7119172.f1.pdf]

CARE case report guidelines

CARE Checklist of information to include when writing a case report

(cc) BY-NC-ND

Topic	Item	Checklist item description	Reported on Line
Title	1	The diagnosis or intervention of primary focus followed by the words "case report"	Title page, paragraph 1
Key Words	2	2 to 5 key words that identify diagnoses or interventions in this case report, including "case report"	Abstract, paragraph 2
Abstract (no references)	3a	Introduction: What is unique about this case and what does it add to the scientific literature?	Abstract, paragraph 1
	3b	Main symptoms and/or important clinical findings	Abstract, paragraph 2
	3c	The main diagnoses, therapeutic interventions, and outcomes	Abstract, paragraph 2
	3d	Conclusion—What is the main "take-away" lesson(s) from this case?	Abstract, pragraph 3
Introduction	4	One or two paragraphs summarizing why this case is unique (**may include references**)	Background, paragraph 1 to 3
Patient Information	5a	De-identified patient specific information.	Case presentation, paragraph 1
	5b	Primary concerns and symptoms of the patient.	Case presentantion, paragraph 1
	5c	Medical, family, and psycho-social history including relevant genetic information	Case presentantion, paragraph 1
	5d	Relevant past interventions with outcomes	Case presentantion, paragraph 2
Clinical Findings	6	Describe significant physical examination (PE) and important clinical findings.	Case presentantion, paragraph 2
Timeline	7	Historical and current information from this episode of care organized as a timeline	N / A
Diagnostic Assessment	8a	Diagnostic testing (such as PE, laboratory testing, imaging, surveys).	Case presentantion, paragraph 2
	8b	Diagnostic challenges (such as access to testing, financial, or cultural)	N / A
	8c	Diagnosis (including other diagnoses considered)	Case presentantion, paragraph 2
	8d	Prognosis (such as staging in oncology) where applicable	Case presentantion, paragraph 2
Therapeutic Intervention	9a	Types of therapeutic intervention (such as pharmacologic, surgical, preventive, self-care)	Case presentantion, paragraph 2
	9b	Administration of therapeutic intervention (such as dosage, strength, duration)	Case presentantion, paragraph 2 to 6
	9c	Changes in therapeutic intervention (with rationale)	Case presentantion, paragraph 5
Follow-up and Outcomes	10a	Clinician and patient-assessed outcomes (if available)	Case presentantion, paragraph 7
	10b	Important follow-up diagnostic and other test results	Case presentantion, paragraph 7
	10c	Intervention adherence and tolerability (How was this assessed?)	Case presentantion, paragraph 7
	10d	Adverse and unanticipated events	N / A
Discussion	11a	A scientific discussion of the strengths AND limitations associated with this case report	Discussion, paragraphs 1 to 7
	11b	Discussion of the relevant medical literature **with references**.	Discussion, paragraph 1 to 7
	11c	The scientific rationale for any conclusions (including assessment of possible causes)	Discussion, paragraph 1 to 7
	11d	The primary "take-away" lessons of this case report (without references) in a one paragraph conclusion	Discussion, paragraph 7
Patient Perspective	12	The patient should share their perspective in one to two paragraphs on the treatment(s) they received	N / A
Informed Consent	13	Did the patient give informed consent? Please provide if requested ... Case presentantion, paragraph 3	Yes ☑ No ☐
